# Spectrum of cardiac diseases among young and older adults defined by echocardiography at Jakaya Kikwete Cardiac Institute: A prospective cross-sectional study

**DOI:** 10.1186/s12872-023-03367-9

**Published:** 2023-07-18

**Authors:** Irene Abela Jonathan, Engerasiya Kifai, Pilly Chillo

**Affiliations:** 1 School of Medicine, Department of Internal Medicine, Muhimbili University of Health and Allied Sciences, Dar es salaam, P.O. BOX 65001, Tanzania; 2 Department of Adult Cardiology, Jakaya Kikwete Cardiac Institute, Dar Es Salaam, Tanzania

**Keywords:** Spectrum, Cardiovascular diseases, Young adults, Echocardiography

## Abstract

**Background:**

Cardiovascular diseases (CVDs) are a major cause of morbidity and mortality worldwide, with data showing an increasing trend. Previously uncommon, CVDs of lifestyle are now increasing in many Sub-Sahara African (SSA) countries including Tanzania. The study aimed at determining the spectrum and distribution of CVDs among young (< 45 years) and older (≥ 45 years) adults referred for echocardiography at Jakaya Kikwete Cardiac Institute (JKCI).

**Methods:**

Hospital-based cross sectional study was conducted among adult patients referred for echocardiography at JKCI between July and December 2021. Patient’s socio-demographic and clinical characteristics were recorded. CVD diagnoses were made using established diagnostic criterias. Comparisons were done using chi-square test and student’s t-test. Multivariable logistic regression analysis was used to determine factors associated with abnormal echocardiography. A significance level was set at p-value < 0.05.

**Results:**

In total 1,050 patients (750 old and 300 young adults) were enrolled. The mean ± SD age was 62.2 ± 10.4 years and 33.5 ± 7.4 years for older and young adults respectively. Hypertension was the commonest indication for echocardiography both in the young (31%) and older (80%) adults. Majority of older adults were found to have abnormal echocardiography (90.7%), while only 44.7% of the young adults had abnormal echocardiography (p < 0.001). For the older adults, the commonest diagnoses were HHD (70.3%), IHD (9.7%), and non-ischemic cardiomyopathy (6.1%) while for young adults, HHD (16.7%), non-ischemic cardiomyopathy (8%), RHD (8%) and MVP (4.3%) were the commonest. The differences in the echocardiographic diagnoses between young and older adults were statistically significant, p < 0.001. Being an older adult, hypertensive, overweight/obese were independently associated with abnormal echocardiography (p < 0.01).

**Conclusion:**

Hypertensive heart disease is the most common diagnosis among adult patients referred for echocardiography at JKCI, both in young and older adults. Primary prevention, early detection and treatment of systemic hypertension should be reinforced in order to delay or prevent its complications.

## Introduction

Worldwide, cardiovascular diseases (CVDs) remain an important cause of morbidity and mortality, with data showing that about two thirds of deaths that occurred in 2019 were due to non-communicable diseases: mainly cardiovascular [1]. Available data also shows that most of these cardiovascular deaths occur in middle and low-income countries [1]. Additionally, current projections show that the major increase of CVDs burden will occur in the developing countries [2].

CVDs are the second most common cause of adult deaths in Sub-Saharan Africa (SSA), as well as a major cause of chronic illness and disability. Moreover half of CVD deaths occur among people aged 30 to 69 years, which is on average ten years younger than in developed countries [3]. While the region is faced with an increase in CVDs due to lifestyle and urbanization, there is still a huge burden of cardiac diseases due to poverty, malnutrition and infectious diseases, and in addition cardiac diseases related to Human Immunodeficiency Virus (HIV) infection and its treatment [4].

A recent systematic review and meta-analysis of 22 studies from Africa (1999–2017) including 10,098 patients found hypertensive heart disease (39.2%) to be the commonest cause of heart failure, followed by cardiomyopathies (21.4%), and rheumatic heart disease (14.1%), while ischemic heart disease was reported to be rare (7.2%) [5]. Similar findings were observed in a study in Tanzania by Makubi et al. in 2014 [6].

The spectrum of CVDs varies between and within countries depending on the stages of epidemiological transition and cardiovascular risk factor profiles. Majority of studies in SSA and Tanzania have reported the spectrum of cardiac diseases among heart failure patients, and with limited number of participants. This study, therefore, aimed at determining the spectrum of cardiac diseases in a large population sample of young and older adults referred for echocardiography at Jakaya Kikwete Cardiac Institute.

## Methods

### Study design, duration, setting and participants

This was a hospital based prospective cross sectional study of adult patients (≥ 18 years) referred for echocardiography examination between July and December 2021 at JKCI. JKCI is a national tertiary level hospital which receives patients from all regions of United Republic of Tanzania referred by regional and zonal referral hospitals for investigation and treatment of cardiovascular diseases. The echocardiography laboratory is situated within the hospital and operates 6 days a week from Monday to Saturday.

### Sample size and sampling method

We used a single proportion formula (N = Z^2^p (1-p)/e^2^) to estimate the minimum required sample size, where N is the minimum required sample size, p is the estimated proportion of abnormal echocardiography (68.8%) from a study by Dominick et al. [7], e is the margin of error or precision (3%) and Z is the standard normal deviation (1.96) corresponding to a 95% confidence interval. After adjusting for a 10% proportion of non-response, the minimum sample size was 1,017. Participants were consecutively enrolled to obtain the required sample size.

### Data collection

A structured questionnaire was used to gather information on socio-demographic as well as clinical characteristics of patients, including age, sex, marital status, education level, indication for echocardiography, co-morbidities and area of residency. Blood pressure was measured using an automated digital sphygmomanometer (model: RAK289, Shenzhen Technology Company, Shenzhen, China) with the patient in seated position. The average of two readings taken at least 5 min apart was recorded as the patient’s blood pressure. Hypertension was defined according to European Society of Cardiology (ESC) and European Society of Hypertension (ESH) 2018 guideline as office SBP values ≥ 140mmHg and/or DBP values ≥ 90mmHg [8].

Patient’s body weight (in kg) was taken using a well-calibrated weighing scale (model: CAS DB-1 H), with the patient wearing no shoes or heavy clothing, height (in cm) was taken using a stadiometer and recorded to the nearest centimeter. Height and weight were used to calculate body mass index (BMI) using the formula: weight (kg)/height (m^2^). Obesity was defined as BMI ≥ 30 kg/m^2^.

All recruited patients underwent echocardiography examination performed by cardiologists following the American Society of Echocardiography guidelines [9] using a Siemens Acuson machine. Echocardiography included parasternal long axis views of the left ventricle and the left atrium as well as four-chamber views of the left ventricle and left atrium and short axis view. Left ventricular internal diameters were measured in 2-dimensional guided M-mode images at the end of diastole. Left atrial size was measured in 2-dimensional guided M-mode images during end systole. Left ventricular ejection fraction was measured using the biplane method of disks (modified Simpson’s rule) in 2-dimensional parasternal long axis view. Other parameters recorded included diastolic function parameters, dimensions of inter-ventricular septum, right ventricular systolic pressure, left atrial volume, and evidence of any Valvular pathology or congenital heart defects. All images were stored in the echocardiogram machine hard disc for later re-reading and all ECHO findings were reviewed by two senior cardiologists. Diagnosis of different cardiac diseases was made based on clinical presentation, underlying conditions (i.e. hypertension and diabetes mellitus) and established standard echocardiographic diagnostic criteria.

### Definitions

**HHD**: Was diagnosed in a known/or new hypertensive patient with concentric/eccentric left ventricular hypertrophy (LVH) or concentric left ventricular remodeling, left atrial dilatation and/ or systolic and/ or diastolic left ventricular dysfunction with neither valve disease nor segment wall motion abnormalities [10]. **LVH**: Was defined as an increased left ventricular mass index (LVMI) to greater than 95 g/m^2^ and 115 g/m^2^ in women and men respectively [11].**Concentric hypertrophy**: Was defined as increased LVMI and a relative wall thickness (RWT) ≥ 0.42; while **eccentric hypertrophy** was defined as increased LVMI and RWT < 0.42 and concentric remodelling defined as normal LVMI with a RWT ≥ 0.42 [11].

**Degenerative Valvular heart disease and other non-rheumatic Valvular lesions** were diagnosed in accordance with the ESC guidelines on management of VHDs [12]. **RHD**: Was diagnosed following the 2012 World Heart Federation criteria for the echocardiographic diagnosis of RHD [13].

#### Dilated cardiomyopathy

Was defined as dilated heart chambers with hypokinesia and ejection fraction less than 45% without any underlying condition [14].

#### Ischemic heart disease

Was diagnosed based on ventricular dysfunction with segmental hypo- or akinesia which could be attributed to a specific coronary artery with or without typical ECG-findings [15].

#### Pericardial effusion

Presence of echo free space between visceral and parietal pericardium [16].

#### TB-pericarditis

Pericardial effusion in a patient with clinically suspected or microbiologically confirmed TB[16].

#### Congenital heart disease

Was diagnosed based on the echocardiographic evidence of a heart defect [17].

#### Cor-pulmonale

Dilated and hypertrophied right ventricle (RV), evidence of increased RV systolic pressure (D-shaped LV in diastole or diastolic flattening of the LV septum) [18].

#### Myocarditis

Was diagnosed in accordance with the European Society of Cardiology guideline for diagnosis and management of pericardial and myocardial diseases [19].

### Data analysis

Data was analysed using SPSS for windows version 23. Data are presented as mean ± SD for continuous variables such as age, SBP, DBP and BMI and as percentages for categorical variables. The proportion of abnormal echocardiography is expressed as percentage. Comparisons between young (< 45 years) and older (≥ 45) adults were done by chi-square test for categorical variables and student’s t-test for continuous variables. Multivariable logistic regression was used to determine factors associated with a finding of abnormal echocardiography. A p-value of < 0.05 was considered statistically significant.

### Ethical consideration

#### Ethical approval

to conduct the study was obtained from Muhimbili University of Health and Allied Sciences’ research and publication committee. Permission to conduct the study was obtained from JKCI management. Written informed consent was obtained from all participants.

## Results

### Study flow diagram

**Fig. 1:**
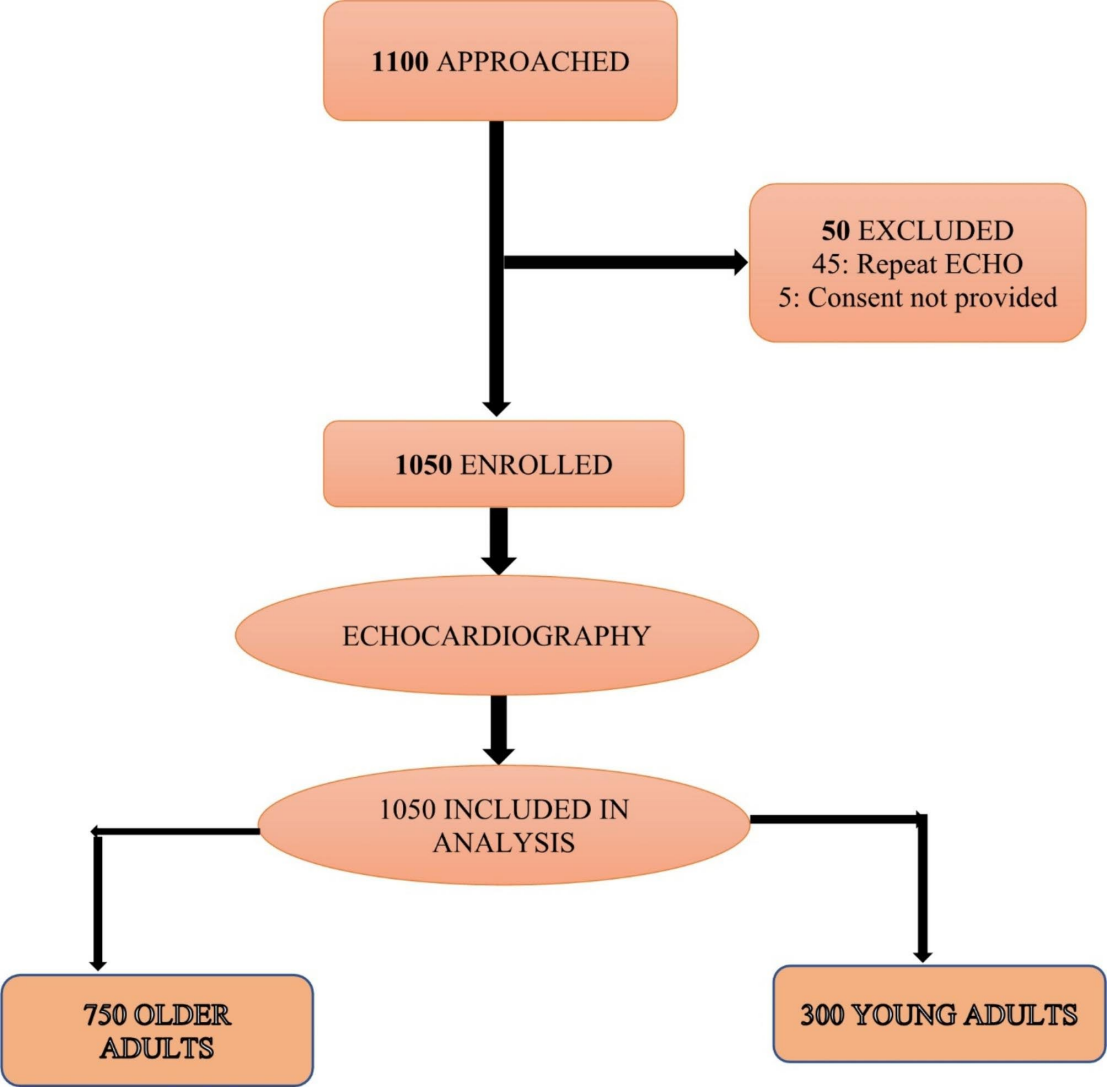
Study flow diagram

During the study period, among patients referred for echocardiography at JKCI, 1100 were approached for consent to participate in this study. Of those approached, 50 were excluded due to reasons shown in Fig. 1.

### Socio-demographic and clinical characteristics

A total of 1050 patients were included in this study. The overall mean ± SD age was 54 ± 16.2 years and was 33.5 ± 7.4 years and 62.2 ± 10.4 years for young and older adults respectively. Females constituted 56.4%, of the overall study population. Over half (57.7%) of young adults had no pre-existing co-morbidity, while 82.1% and 16% of older adults had a history of hypertension and diabetes mellitus respectively, with 10.6% having more than one co-morbidity. Table 1.

**Table 1:** Socio-demographic and clinical characteristics of study participants (N = 1050)

Variable	Total population N = 1,050	Young n = 300	Older n = 750	*P*-value
**Mean ± SD age (years)**	54 ± 16.1	33.5 ± 7.4	62.2 ± 10.4	0.003
***Sex, n (%)***				
Females	592 (56.4)	171 (57)	421 (56.1)	0.79
***Education level, n (%)***				
No formal education	71 (6.8)	6 (2)	65 (8.7)	< 0.001
Primary	199 (19.0)	55 (18.3)	144 (19.2)	0.75
Secondary	433 (41.2)	145 (48.3)	288 (38.4)	0.003
College/University	347 (33.0)	94 (31.3)	253 (33.7)	0.46
***Residence, n (%)***				
Dar es Salaam	643 (61.2)	188 (62.7)	455 (60.7)	0.55
Up-country	407 (38.8)	112 (37.3)	295 (39.3)	0.55
***Medical History, n (%)***				
History of HTN	716 (68.2)	100 (33.3)	616 (82.1)	< 0.001
History of DM	128 (12.2)	8 (2.7)	120 (16)	< 0.001
***Blood pressure findings***				
Mean ± SD, SBP (mmHg)	137 ± 26	125 ± 22	142 ± 26	< 0.001
Mean ± SD, DBP (mmHg)	81 ± 15	79 ± 16	82 ± 15	0.003
Raised BP at enrollment, n (%)	517 (49.2)	98 (32.7)	419 (55.9)	< 0.001
Known hypertensive with normal BP at enrollment, n (%)	268 (37.4)	37 (37)	231 (37.5)	0.54
***Anthropometric measurements***				
Mean ± SD, Height, (cm)	160.9 ± 32.5	161.2 ± 8.3	160.8 ± 38.1	0.79
Mean ± SD, Weight, (kg)	76.6 ± 16.8	70.9 ± 16.9	78.9 ± 16.3	< 0.001
Mean ± SD, BMI, (kg/m^2^)	29.9 ± 6.5	27.3 ± 6.3	31.0 ± 6.3	< 0.001
***BMI status, n (%)***				
Normal	236 (22.5)	126 (42)	110 (14.6)	< 0.001
Overweight	326 (31%)	87 (29%)	239 (31.9%)	0.35
Obese	488 (46.5%)	87 (29%)	401 (53.5%)	< 0.001

**SD**: Standard deviation, **HTN**: Hypertension, **DM**: Diabetes mellitus, **SD**: Standard deviation, **SBP**: Systolic blood pressure, **DBP**: Diastolic blood pressure, **BMI**: Body mass index

### Indications for echocardiography

**Fig. 2:**
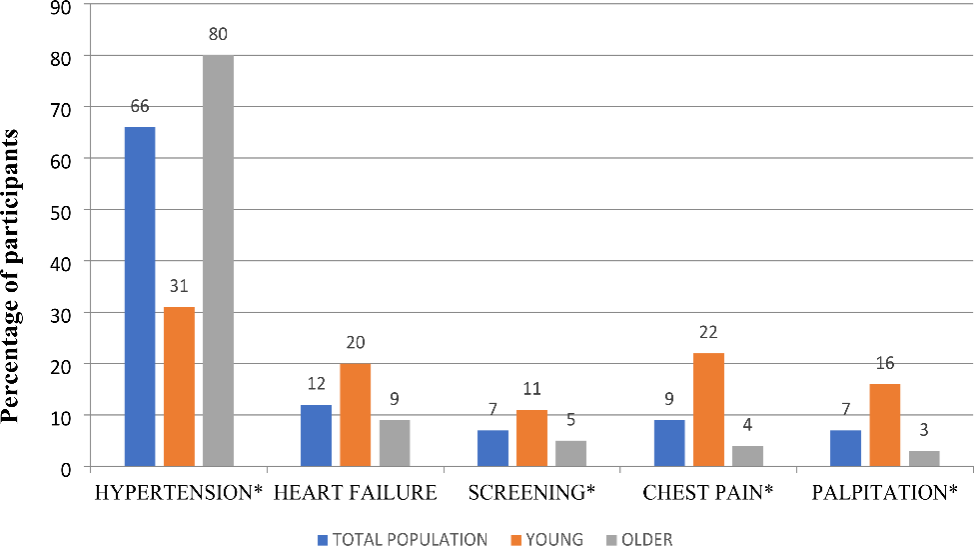
Indications for echocardiography in the total population and among young and older adults (N = 1050) *Indicates comparisons that were statistically significant (p < 0.001)

The main clinical indication for echocardiography overall and in older adults was hypertension (79.7%) while chest pain, palpitations and screening (pre-operative and pre-employment reasons) were common indications in young adults. Other less common indications were syncope and stroke (not included in figure below), Fig. 2.

### Proportion of abnormal echocardiography

Overall, abnormal echocardiography was found in 814 (77.5%) patients. According to age groups abnormal echocardiography was found in 134 (44.7%) and 680 (90.7%) young and older adults respectively and the difference was statistically significant (p < 0.001).

### Pattern/types of cardiac diseases among patients referred for echocardiography

**Fig. 3:**
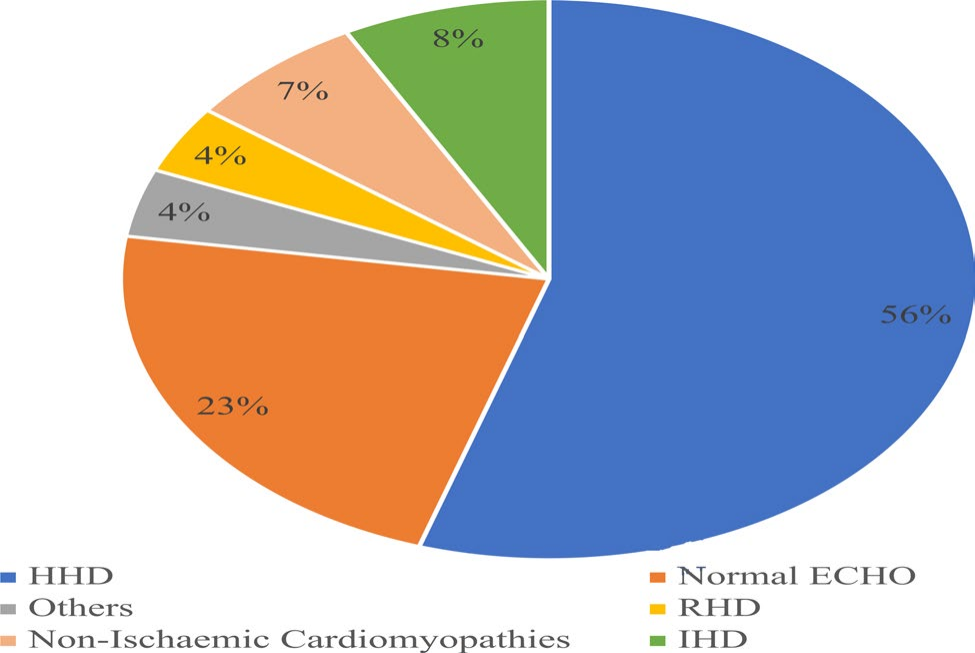
Pattern of echocardiographic diagnosis (N = 1050) ***HHD***: *Hypertensive heart disease*, ***IHD***: *Ischaemic heart disease*, ***RHD***: *Rheumatic heart disease.****OTHERS***: *Mitral valve prolapse (1.4%), Cor-pulmonale (1%), Degenerative Valvular heart disease (0.6%), Pericardial diseases (0.6%), Adult congenital heart diseases (0.6%-Congenitally corrected transposition of great arteries, ventricular septal defect, atrial septal defect with partial anomalous pulmonary venous return and tetralogy of fallot), Left atrial myxoma (0.2%) and myocarditis (0.2%).*

In the total population HHD was the most common echocardiographic diagnosis (55.5%), followed by IHD (7.7%), non-ischemic cardiomyopathy (6.7%), and rheumatic heart disease (3.9%). Out of the 70 non-ischemic cardiomyopathies 53 (78.6%) were idiopathic dilated cardiomyopathy, 9 (12.9%) were peripartum cardiomyopathy, and the remainder 6 (8.5%) were restrictive cardiomyopathies. Other diagnoses are as seen in Fig. 3.

### Distribution of different echocardiographic diagnosis by age groups

Young adults were more likely to have normal echocardiography (55.3% versus 9.3%; p < 0.001), RHD (8% versus 2.3%, p < 0.001), MVP (4.3% versus 0.3%, p < 0.001), pericardial diseases (1.3% versus 0.3%, p = 0.038), CHD (100%, p = 0.002), and myocarditis (100%, p = 0.025), compared to older adults. While, HHD (70.3% versus 16.7%, p < 0.001), and IHD (9.7% versus 2.7%, p < 0.001) were common in older adults compared to their younger counterpart, Table 2.

**Table 2:** Distribution of different echocardiographic diagnosis by age groups

Diagnosis n (%)	Young adults n = 300	Older adults n = 750	*P* Value
Normal ECHO	166 (55.3)	70 (9.3)	< 0.001*
HHD	50 (16.7)	527 (70.3)	< 0.001*
IHD	8 (2.7)	73 (9.7)	< 0.001*
Non- ischemic cardiomyopathy	24 (8)	46 (6.1)	0.27
RHD	24 (8)	17 (2.3)	< 0.001*
MVP	13 (4.3)	2 (0.3)	< 0.001*
Cor-pulmonale	3 (1)	7 (0.9)	0.92
Degenerative VHD	1 (0.3)	5 (0.7)	0.52
Pericardial diseases	4 (1.3)	2 (0.3)	0.04*
Adult CHD	4 (1.3)	0 (0.0)	0.002*
LA myxoma	1 (0.3)	1 (0.1)	0.50
Myocarditis	2 (0.7)	0 (0.0)	0.03*

**MVP**: Mitral valve prolapse, **RHD**: Rheumatic heart disease, **VHD**: Valvular heart disease, **CHD**: Congenital heart disease, **LA**: Left atrium, **HHD**: Hypertensive heart disease, **IHD**: Ischaemic heart disease,

* Statistically significant p- values

### Socio-demographic and clinical predictors of abnormal echocardiography

Being an older adult, hypertensive as well as being overweight or obese was independently associated with a finding of abnormal echocardiography (p < 0.01), Table 3.

**Table 3:** *Socio-demographic and clinical predictors of abnormal echocardiography: Logistic regression for univariate and multivariate analysis (N = 1050)*

Factor	Univariate analysis COR (95%CI)	*p*-value	Multivariate analysis aOR (95% CI)	*p*-value
**Age groups**				
Young	Ref		Ref	
Older	12 (8.6–16.8)	< 0.001	7.5 (5.1–11.0)	< 0.001*
**Hypertension**				
Yes	8.6 (6.2–11.9)	< 0.001	4.6 (3.2–6.7)	< 0.001*
No	Ref		Ref	
**Diabetes mellitus**				
Yes	4.9 (2.4–10.2)	< 0.001	2.2 (0.9–4.8)	0.06
No	Ref		Ref	
**BMI status**				
Normal	Ref		Ref	
Overweight & obese	1.9 (1.3–2.6)	< 0.001	1.3 (1.2–2.8)	0.01*
**Residence, n (%)**				
Upcountry	1.3 (0.9–1.8)	0.082	1.4 (0.9–1.9)	0.10
Dar es Salaam	Ref		Ref	

**BMI**: Body Mass Index, **ECHO**: Echocardiography, * statistically significant p- values

## Discussion

We sought to describe the spectrum of cardiac diseases among young and older adult patients referred for echocardiography at Jakaya Kikwete Cardiac Institute. Hypertension accounted for over half of the co-morbidities among these patients. Proportionally abnormal echocardiography was found in more than two-third of the participants, with the commonest diagnosis being hypertensive heart disease, followed by ischemic heart disease, non-ischemic cardiomyopathies and rheumatic heart disease.

Hypertensive heart disease was the commonest overall echocardiographic diagnosis in both young and older adults accounting for 55.5% of all diagnoses, this finding is consistent with multiple studies in different parts of Africa, including those in Cameroon [20–23], Nigeria [24–26], the TaHeF study [6] and a study by Dominick et al. in Tanzania [7]. These are also similar to the findings of the SSA survey of heart failure [27] which showed that HHD was the predominant cause of heart failure accounting for 45.4%. These consistent findings could be explained by the common fact that majority of participants included in these studies had hypertension as a co-morbidity. Furthermore, the present finding of HHD in young adults may reflect an epidemiological shift, a late detection and or poorly treated hypertension since previous studies done in SSA show that awareness of hypertensive status is low and that less than 20% of the hypertensive patients are within therapeutic targets [28, 29].

Ischemic heart disease was second to HHD in the overall population and in older adults constituting 7.7%, while it was least common in young adults. The finding of IHD being second to HHD is contrary to previous studies done in Africa, which found non-ischemic cardiomyopathies as the second commonest etiology with IHD being the least [6, 25, 30]. However proportionally it was similar to findings from the SSA survey of heart failure (7.7%). These differing findings can be explained by older age of the study population and tertiary/referral nature of the study site which could entail better diagnostics and expertise for making the diagnosis, while those with predominance of non-ischemic cardiomyopathy had a relatively younger study population and majority were conducted in North eastern and western Nigeria [25, 26], where peripartum cardiomyopathy is higher compared to other parts of Africa hence contributing significantly to non-ischemic cardiomyopathies. Moreover, these findings in totality may reflect an epidemiological transition from diseases of poverty and infections to life-style related diseases.

Non-ischemic cardiomyopathies were the third most common diagnosis in both young and older adults with dilated cardiomyopathy being the most common type of cardiomyopathy. Similar findings were found in three studies done in Cameroon [20, 22, 23], a study done in Nigeria [24], and that by Dominick et al. in rural Tanzania [7] where non-ischemic cardiomyopathy was third to HHD and VHD. However these findings differ from studies by Makubi et al., Clovis et al., Mohamed T, and Hadiza saidu et al. [6, 23, 25, 30], which found non-ischemic cardiomyopathy as the second most common diagnosis. The differences can be explained by the fact that the mean age of our study population was 54, relatively older, 80.4% had co-morbidities (HTN and DM), and 80% were overweight or obese, these all increasing the risk for HHD and IHD rather than non-ischemic cardiomyopathy.

Rheumatic heart disease (3.9%) was the fourth common diagnosis in overall population but the second most common in young adults with mitral regurgitation being the predominant Valvular pathology (44%). These findings showing the least contribution of RHD in the overall population are consistent to the findings from the TaHeF study [6], study done in Cameroon by Ahmadou et al. [22], and a study by Hadiza Saidu in Nigeria [25]. In young adults RHD remain to be a common diagnosis similarly to the findings form a study done in Nigeria [24], Cameroon [22], rural Tanzania by Dominick et al. [7]. However, on the contrary studies done in Ethiopia [31, 32], found RHD to be the commonest overall echocardiographic diagnosis. These differences can be explained by the tertiary/urban nature of our study setting while those in Cameroon were conducted in rural settings where rheumatic heart disease is most prevalent.

In this study, older age group, presence of comorbidities (hypertension and DM), being overweight or obese and undergoing echocardiography for an indication other than screening were found to be significantly associated with an abnormal echocardiography, with older age group, co-morbidities and obesity being independent predictors. These findings echoes the already existing knowledge on the traditional cardiovascular risk factors as documented in previous studies [27, 33–36].

This study provides the current spectrum and documents the distribution of cardiac diseases in young and older adults in a large sample of patients irrespective of their heart failure status compared to other studies. However, the study is limited by cross-sectional nature hence temporal relationship of different cardiac diseases cannot be established, the generalizability to the community and lower-level facilities cannot be done since it was conducted at a tertiary hospital. Furthermore, the study included echocardiographic data only, hence the contribution of arrhythmias and conduction abnormalities in the spectrum of cardiac diseases is lacking and the diagnosis of ischemic heart disease were made based on clinical presentation and echocardiography findings which makes these presumptive since coronary angiography was not done to show evidence of occlusion.

## Conclusion

Life-style related CVDs are becoming predominant with HHD being the commonest. Therefore, efforts should be directed towards prevention, early detection and appropriate management of hypertension in order to prevent or delay its complications.

## Data Availability

The datasets generated and analysed during the current study are available from the corresponding author on reasonable request.

## Declarations

**Ethics approval and consent to participate**.

Written informed consent was obtained from the study participants. The study was approved by the local ethics committee of Muhimbili University of Health and Allied Sciences’ Ethical Review Board. Permission to do the study was obtained from Jakaya Kikwete Cardiac Institute management. All methods were carried out in accordance with relevant guidelines and regulations.

**Consent for publication**.

## Abbreviations

**BMI**: Body Mass Index
**CHD**: Congenital Heart Disease
**CVDs**: Cardiovascular diseases
**DBP**: Diastolic Blood Pressure
**DCM**: Dilated cardiomyopathy
**ECHO**: Echocardiography
**ESC**: European Society of Cardiology
**HF**: Heart failure
**HHD**: Hypertensive Heart Disease
**HTN**: Hypertension
**IHD**: Ischaemic Heart Disease
**JKCI**: Jakaya Kikwete Cardiac Institute
**LVH**: Left Ventricular Hypertrophy
**MUHAS**: Muhimbili University of Health and Allied Sciences
**MVP**: Mitral Valve Prolapse
**NCDs**: Non-communicable diseases
**RHD**: Rheumatic Heart Disease
**SBP**: Systolic Blood Pressure
**SSA**: Sub-Saharan Africa
**VHD**: Valvular Heart Disease
